# Protocol for autofluorescence-driven isolation of human peripheral blood eosinophils

**DOI:** 10.1016/j.xpro.2024.103451

**Published:** 2024-11-19

**Authors:** Tobias Weihrauch, Natalie Gray, N. Helge Meyer, Karin Loser, Ulrike Raap

**Affiliations:** 1 Division of Experimental Allergy and Immunodermatology, School of Medicine and Health Sciences, Carl von Ossietzky University Oldenburg, Oldenburg, Germany; 2 Division of Anatomy, School of Medicine and Health Sciences, Carl von Ossietzky University Oldenburg, Oldenburg, Germany; 3 University Hospital of General and Visceral Surgery, Department of Human Medicine, University of Oldenburg and Klinikum Oldenburg, Oldenburg, Germany; 4 Division of Immunology, Faculty of Medicine and Health Sciences, University of Oldenburg, Oldenburg, Germany; 5 Research Center for Neurosensory Science, Carl von Ossietzky University Oldenburg, Oldenburg, Germany; 6 University Clinic of Dermatology and Allergy, University of Oldenburg, Oldenburg, Germany

**Keywords:** Cell separation/fractionation, Flow Cytometry, Immunology

## Abstract

Most common techniques for isolating eosinophils utilize CD16-negative selection, neglecting the CD16-positive fraction of eosinophils. Here, we present a protocol for isolating human CD16+ and CD16− eosinophils based on their autofluorescence using the MACSQuant Tyto cell sorter. We describe steps for purifying eosinophils and assessing purity, viability, and functional activity. We then detail procedures for Giemsa staining and the assessment of CD16 expression on human eosinophils. This protocol yields eosinophil purities of over 99%.

## Highlights

• Instructions for purifying eosinophils based on autofluorescence using cell sorting
• Steps to assess eosinophil purity and viability by flow cytometry and Giemsa staining
• Guidance on performing an eosinophil activation test to confirm functional activity
• Manual for analyzing CD16 expression on purified eosinophils

## Abstract (editor highlights)

Publisher’s note: Undertaking any experimental protocol requires adherence to local institutional guidelines for laboratory safety and ethics.

## Graphical abstract

**Figure:**
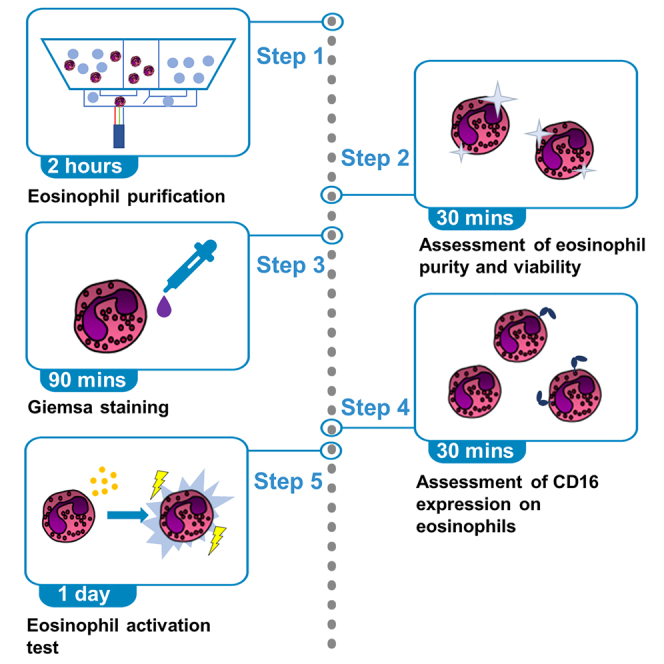

## Before you begin

For investigating eosinophils, the cells are usually isolated from peripheral blood with methods such as immunomagnetic selection or density gradient centrifugation. However, these techniques, especially density gradient centrifugation, have the potential of resulting in lower yields or viability.^1^ In the case of immunomagnetic selection these parameters may also be influenced as a result of manufacturer specific isolation kit composition and the mode of immunomagnetic selection.^2^ In addition, negative immunomagnetic selection of eosinophils commonly excludes CD16+ (FcγRIII) eosinophils, though this minor cell population is implicated in health and disease. For allergic conditions such as allergic rhinosinusitis or asthma, increased CD16 expression on eosinophils has been described when compared to eosinophils of non-atopic patients.^3^ Given that eosinophils are a relatively rare type of leukocyte, and experiments usually require hundreds of thousands of cells, it is crucial to isolate them as effectively and gently as possible from patients’ blood. In comparison to other leukocytes, eosinophils show a marked autofluorescence with an emission wavelength of ∼520 nm and local excitation maxima of ∼380 nm and ∼450 nm. This autofluorescence is caused by flavin adenine dinucleotide (FAD), flavin mononucleotide (FMN), and riboflavin in eosinophil granules.^4^ Based on this unique characteristic, eosinophils can be isolated from other leukocytes using a completely label-free method, without relying on techniques that include antibodies, such as negative immunomagnetic selection.

This protocol presents a highly effective and cell-friendly method for autofluorescence-dependent isolation of eosinophils, including CD16+ eosinophils, from venous blood foregoing the necessity of antibodies or beads. The isolation of eosinophils is performed using the MACSQuant Tyto cell sorter. We consistently get a high purity of > 99% and an average viability of 93% with this time-efficient method. Therefore, we further describe the procedures of determining these percentages in addition to visualizing the purity by Giemsa staining. In order to assess CD16− and CD16+ eosinophil populations, we provide a CD16 staining protocol. To show that the eosinophils are not pre-activated and cell activation is still inducible after sorting we performed an eosinophil activation test. We used some of the supporting methods recently for investigating TRPV1 (Transient Receptor Potential Vanilloid 1) on human eosinophils.^5^ However, this isolation technique can be used for any subsequent experiments with human eosinophils, especially when investigating differences between health and disease due to the inclusion of CD16+ eosinophils.

### Institutional permissions

The study was conducted in accordance with the Declaration of Helsinki and approved by the Ethics Committee of Carl von Ossietzky University Oldenburg (protocol code 2021–025) for studies involving humans. Patient materials were collected by the Department of Dermatology and Allergy of Human Medicine at the Klinikum Oldenburg.

Before performing the following protocol with blood samples, make sure to get an approval by your local ethics committee.

### Biosafety

The work with potentially infectious material such as human blood or purified cells requires proper biosafety practices. We recommend wearing personal protective equipment such as lab coat, gloves, and eye protection, and using filter tips for pipettes. The conscientious decontamination of all reagents and surfaces after finishing the experiments is essential. We recommend autoclaving all waste products and using commercial aldehyde-free decontamination solutions (e.g., Inciding Plus or Lerasept), according to the manufacturer’s instructions, to decontaminate surfaces.

### Collection of blood samples

**Timing: 10 min**

1. Obtain blood by drawing blood via venipuncture into monovettes that have been preloaded with the appropriate amount of Ethylenediaminetetraacetic acid (EDTA) to obtain a final concentration of 1.6 mg EDTA per mL of blood.
  a. Pull back the plunger gently to minimize force that could potentially damage the cells.
  b. Invert the monovettes five times carefully to ensure the blood mixes well with the EDTA.

### Preparation of devices and reagents

**Timing: 30 min**

2. Start and initialize all devices and precool the centrifuges and MACSQuant Tyto to 4°C.
3. Prepare the reagents as listed in materials and equipment.

## Key resources table

**Table:** 

REAGENT or RESOURCE	SOURCE	IDENTIFIER
**Antibodies**		

APC anti-human CD69 antibody (undiluted)	Miltenyi Biotec	Cat# 130-112-614; Clone: REA824
APC-A750 anti-human CD16 antibody (undiluted)	Beckman Coulter	Cat# B49184; Clone: 3G8
PE-Cyanin 7 (PC7) anti-human Siglec-8 antibody (undiluted)	BioLegend	Cat# 347112; Clone: 7C9 RRID: AB_2629720

**Biological samples**		

Human venous blood, freshly drawn	Klinikum Oldenburg	N/A

**Chemicals, peptides, and recombinant proteins**		

7-AAD staining solution	Miltenyi Biotec	Cat# 130-11-568
Albumin bovine serum (BSA)	VWR	Cat# 421501J
Buffer tablets pH 7.2	Merck	Cat# 1.09468.0100
Giemsa’s azure eosin methylene blue solution	Merck	Cat# 1.09204.2500
IL-3	PeproTech	Cat# 200-03-2UG
MACS Quant Tyto running buffer	Miltenyi Biotec	Cat# 130-107-207
Methanol	ITW Reagents	Cat# 221091
Neo-Mount mounting medium	Merck	Cat# 1.09016.0500
Penicillin-Streptomycin	Gibco	Cat# 15140122
Phosphate-buffered saline (PBS)	Roth	Cat# 9143.2
RBC Lysis buffer	BioLegend	Cat# 420302
RPMI 1640 medium	VWR	Cat# 392–0426

**Software and algorithms**		

Kaluza 2.1.1	Beckman Coulter	N/A

**Other**		

1.5 mL microcentrifuge tubes	Sarstedt	Cat# 72.690.001
50 mL polypropylene (PP) conical tubes	Sarstedt	Cat# 62.547.254
9 mL EDTA K3E preloaded monovettes	Sarstedt	Cat# 02.1066.001
Adhesive microscopy slides	Marienfeld Superior	Cat# 0810001
Cover slips, 18 mm × 18 mm	VWR	Cat# 631–1567
Cytoflex S	Beckman Coulter	N/A
Disposable syringe (10 mL)	Roth	Cat# EP97.1
Epredia TPX single sample chamber	Fisher Scientific	Cat# A78710018
Filter cards	Fisher Scientific	Cat# 5991022
Fisherbrand Gel-loading tips, 1–200 μL	Fisher Scientific	Cat# 11367801
Galaxy 170S CO_2_ incubator	Eppendorf	N/A
Glass staining trough	Engelbrecht	Cat# 42460
Hydrophobic barrier pen	Merck	Cat# Z377821-1EA
MACSQuant Tyto Cartridge	Miltenyi Biotec	Cat# 130-104-791
MACSQuant Tyto Priming Fixture	Miltenyi Biotec	Cat# 130-119-832
MACSQuant Tyto	Miltenyi Biotec	N/A
Microcentrifuge 5425R	Eppendorf	N/A
MyBlock Mini Dry Bath heat block	Benchmark Scientific	Cat# BSH200-HL
Olympus BX51 Microscope	Olympus	N/A
Pipette tips (10 μL)	Biozym	Cat# VT0290
Pipette tips (200 μL)	Biozym	Cat# VT0240
Pipette tips (1250 μL)	Biozym	Cat# VT0270
Pre-separation filters (20 μm)	Miltenyi Biotec	Cat# 130-101-812
Research Plus pipette (0.5–10 μL)	Eppendorf	Cat# 3123000020
Research Plus pipette (10–100 μL)	Eppendorf	Cat# 3123000047
Research Plus pipette (100–1000 μL)	Eppendorf	Cat# 3123000063
Shandon Cytoclip stainless steel slide clip	Fisher Scientific	Cat# 12608036
Shandon Cytospin 4 cytocentrifuge	Fisher Scientific	Cat# A78300003
Tabletop centrifuge 5810R	Eppendorf	N/A
Vornado Mini Vortex Mixer	Benchmark Scientific	Cat# BV101-B

## Materials and equipment

**Table:** Lysis buffer

Reagent	Final concentration	Amount
RBC Lysis buffer 10x	1x	100 mL
ddH_2_O	N/A	900 mL
**Total**	**N/A**	**1000 mL**

Store at 4°C for up to 3 months.

### PBS + 1% BSA

Dissolve 2 g purified BSA in 20 mL ultrapure water or 1X PBS to obtain a 10% BSA stock solution by mixing in a 50 mL conical tube.

Dilute 5 mL of 10% BSA with 45 mL 1X PBS.

### Staining buffer

Dissolve one buffer tablet (pH 7.2) in 1 L ultrapure water on a magnetic stirrer.

Store at 4°C for up to 3 months.

**Table:** Giemsa staining solution

Reagent	Final concentration	Amount
Giemsa’s azure eosin methylene blue solution	1:20	1 mL
Staining buffer	N/A	19 mL
**Total**	**N/A**	**20 mL**

Prepare freshly and wait 10 min before usage.

## Step-by-step method details

### Isolation of human eosinophils via autofluorescence

**Timing: 2 h**

This protocol outlines a reliable method for isolating eosinophils from whole human blood with the MACSQuant Tyto Cell Sorter, using their autofluorescence signal. However, this procedure can be adapted for use with any other cell sorter that is equipped with a violet laser and utilizes the same or similar wavelength detection channels. For specific details, refer to the instruction manual of your cell sorter.

**Figure 1:**
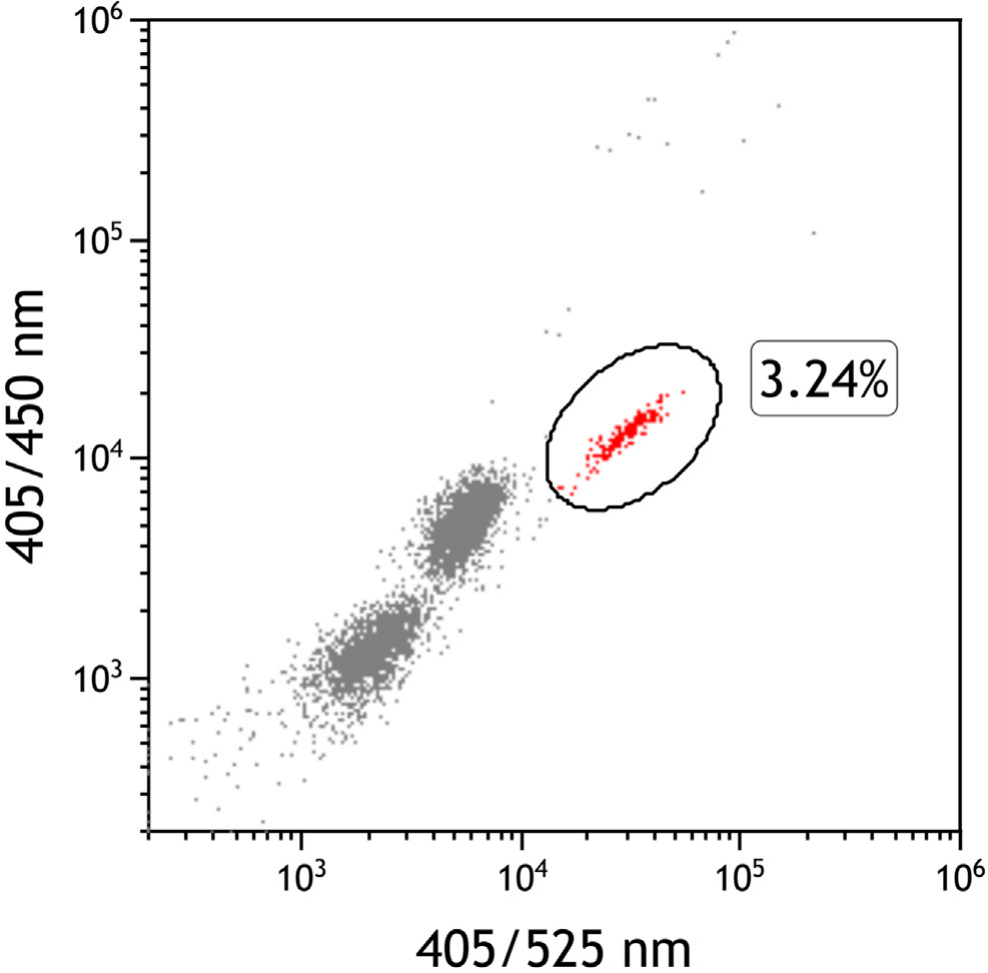
Determining eosinophil percentage after the lysis step Representative dot plot showing unsorted cells after the lysis step on eosinophil autofluorescence-specific wave lengths of 405/450 nm and 405/525 nm. The gate for eosinophils was set on according to the positively separated cell population. Percentage and total cell count were used for the calculation of dilution with MACSQuant Tyto Running Buffer.

1. Red Blood Cell Lysis.
  a. Transfer 3–5 mL of EDTA blood into a 50 mL conical tube, fill up to 50 mL with lysis buffer and vortex the cell suspension for 5 s.
  b. Incubate at 19°C–22°C for 7 min.
  c. Centrifuge the cell suspension at 300 × *g* for 10 min at 4°C.
  d. Decant and discard the supernatant.
    **CRITICAL:** No supernatant should remain in the conical tube, since any debris and dilution of cells prolongs the duration of the eosinophil sorting procedure and raises the sorting volume.
  e. Resuspend the cell pellet in 1 mL PBS + 1% BSA and place the cell suspension on ice.
2. Predetermination of eosinophil cell count and rate.
  a. Dilute 10 μL of cell suspension with 40 μL PBS.
  b. On a Beckman Cytoflex S, eosinophil autofluorescence is best detected at 405/450 nm and 405/525 nm, e.g., corresponding to the PB450/VioBlue or KO525/VioGreen channels. Set the eosinophil autofluorescence gate according to Figure 1.

**CRITICAL:** We used a Cytoflex S (Beckman Coulter) flow cytometer for cell counting. When using other flow cytometry devices, please check the specific settings and use equivalent channels or channels detecting similar wave lengths.

3. Loading cells into the sorting chamber.
  a. To prime the cartridge, place it on the MACS Quant Tyto Priming Fixture by aligning the feet with the slots in the base (Figure 2).

**Figure 2:**
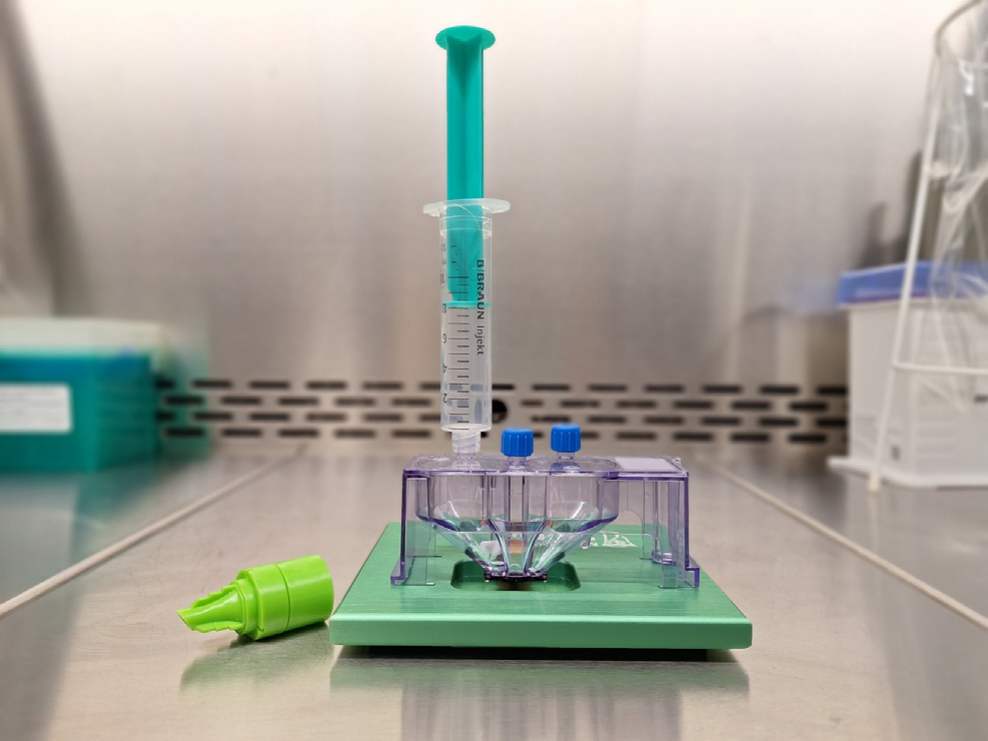
Set-up for cartridge priming The cartridge is placed on the dark green magnetic priming fixture in order to open the sort valve. A Syringe is screwed onto the input chamber. A Pre-Separation Filter (20 μm; green) is used for the following addition of the cell suspension.
  b. Open the intake of the input chamber (chamber with the depiction of an owl).
  c. Attach a syringe of which the plunger has been removed, onto it (Figure 2).
  d. Add 300 μL PBS + 1% BSA into the syringe.
  e. Insert the plunger and slowly and carefully push it down until PBS becomes visible in the positive collection chamber (middle chamber) and stop immediately. Troubleshooting 1.
  f. Remove the cartridge from the MACS Quant Tyto Priming Fixture and close the pressure inlet with the O-ring, which is located at the right corner underneath the cartridge, using your thumb.
  g. Continue pressing the plunger down until PBS becomes visible in the negative collection chamber. Stop immediately and remove the syringe.
    **CRITICAL:** Do not touch the microchip underneath the cartridge at any time. Troubleshooting 2.
  h. Place the cartridge in its plastic packaging on ice and remove the syringe.
  i. Dilute the 1 mL cell suspension in the appropriate calculated amount of Tyto Running Buffer and mix gently.
    **CRITICAL:** Adjust the cell concentration to a maximum of 2.1 × 10^6^ total cells per mL sorting volume with an eosinophil rate between 0.8% and 8.2%.
    **CRITICAL:** Be aware of the maximum sorting volume of 10 mL per sort. A higher volume and dilution of the cell suspension will result in a longer duration of sort but also in higher purities. The maximum predicted eosinophil count must not exceed 1 × 10^6^ cells/mL in the final sorting volume.
  j. Attach a syringe without the plunger onto the intake of the input chamber, place a green 20 μm pre-separation filter (Figure 2) on the syringe and filter the entire cell suspension.
    ***Note:*** If the cell suspension does not flow through the filter automatically, place a thumb flat on the intake of the filter and apply some air pressure onto the filter without touching the cell suspension. Alternatively, the filter can be moisturized with Tyto running buffer before usage.
  k. Remove the pre-separation filter, carefully insert the plunger into the syringe, and transfer the cell suspension into the input chamber.
  l. Remove the syringe and close the intake of the input chamber.
4. Eosinophil sort on MACSQuant Tyto.
  a. Scan the 2D barcode of the cartridge at the front of the MACS Quant Tyto Cell Sorter.
  b. Open the lid of the instrument and insert the cartridge in the correct orientation (with the input chamber facing the user and front of the instrument). The cartridge is placed correctly if the propeller in the input chamber starts spinning.
  c. In the user interface, select the cartridge type and enter the sample name and total sorting volume.
  d. Configure the live dot plot by selecting the channels CFP/VioBlue-H on the Y-axis and VioGreen-H on the X-axis.
  e. Start the cell flow in the cartridge by clicking the Play button in the lower right corner.
  f. Set a gate on the separated eosinophils population according to Figure 3 and select this gate as the sorting gate.

**Figure 3:**
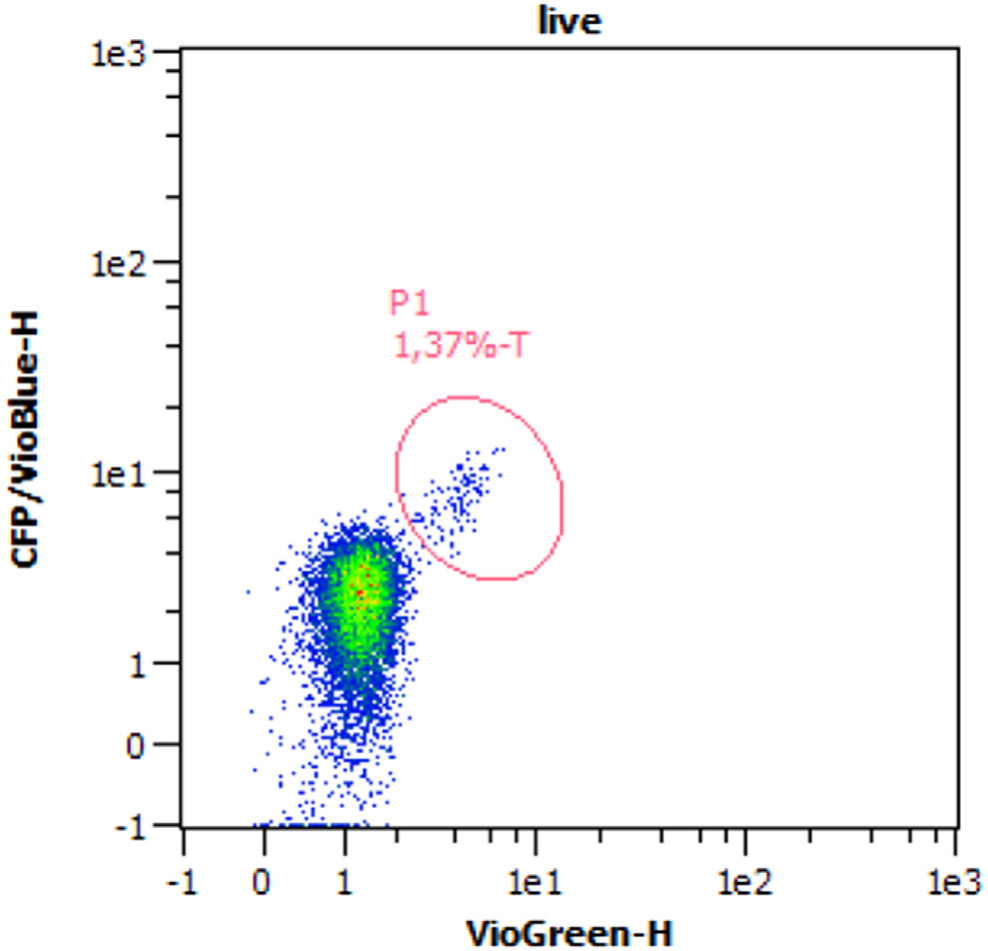
Eosinophil sorting using the MACSQuant Tyto Cell Sorter Representative dot plot of eosinophil sorting with sort gate (P1) on the eosinophil population.
  g. Start sorting by pressing the Sort button in the lower right corner.
    **CRITICAL:** An abortion rate below 1% will result in a high yield and purity. If the abortion rate is higher, verify the purity of sorted eosinophils in order to ensure precise and effective sorting. If eosinophil purity and yield are inadequate, further dilute the cell suspension in the input chamber with Tyto running buffer.
  h. Stop the sort once all cells have been sorted or the desired number of sorted eosinophils has been acquired. Troubleshooting 3.
  i. Open the sort chamber and transfer the sorted eosinophils to a 1.5 mL microcentrifuge tube with a gel-loading tip.
    **CRITICAL:** Measure the exact volume with the pipette for subsequent calculations of eosinophil concentrations.
  j. Remove any remaining eosinophils from the sort chamber by washing it with 100 μL PBS. Collect the washing solution in the microcentrifuge tube and repeat this washing step twice. Keep eosinophils on ice.
  k. The eosinophils are now ready to be used in further experiments.

### Flow cytometry staining to assess eosinophil purity and viability

**Timing: 30 min**

This protocol describes the procedure to analyze eosinophil purity and viability, as calculated from the total number of cells within the sorted fraction, using the eosinophil-specific marker Siglec-8 and DNA-intercalating 7-AAD.

5. Transfer the appropriate volume containing 50,000 sorted eosinophils to a new microcentrifuge tube and centrifuge for 7 min at 350 × *g* and 4°C.
6. Carefully discard the supernatants without disturbing the cell pellet.

***Note:*** Eosinophil pellets might not be visible to the naked eye. Pay attention to the orientation of the microcentrifuge tube in the centrifuge to identify the location of the pellet.

7. Resuspend the eosinophil pellets with 100 μL PBS and mix thoroughly by pipetting.
8. Transfer 50 μL into each of two new 1.5 mL microcentrifuge tubes.
9. Add 1 μL of PC7 conjugated anti-Siglec-8 to one of the tubes and mix thoroughly by pipetting.

**CRITICAL:** If antibodies from other manufacturers are used, it may be necessary to adjust the volume. Thus, we recommend using the antibody listed above.

10. Add 1 μL of 7-AAD staining solution to the other tube and mix gently by pipetting.

**CRITICAL:** A fluorescence minus one (FMO) control should be included to set the gates for marker positive or negative cells. For this purpose, measure without staining for Siglec-8 or 7-AAD in the FMO samples to identify Siglec-8 and 7-AAD negative populations in the flow cytometry dot plot.

11. Incubate both tubes in the dark at 19°C–22°C for 10 min.
12. Dilute both of the stained cell suspensions with 200 μL PBS.
13. Proceed with flow cytometry analysis by measuring the PC7 (Siglec-8) and PC5.5 (7-AAD) fluorescence signals. Troubleshooting 3 and 4.

### Giemsa staining

**Timing: 1.5 h**

This protocol describes the staining technique for distinguishing various cell types. It can also be used to visually assess the purity of isolated human eosinophils in cytospins. The characteristic bilobed nuclei will appear violet, and the granules will appear red. We recently described this protocol for basophils.^6^

14. Preparing the cytospins.
  a. Centrifuge 50,000 purified eosinophils per slide for 7 min at 350 × *g*.
  b. Resuspend the eosinophil pellet with 50 μL PBS.
  c. Place the filter card between the microscopy slide and the sample chamber so that the hole in the card aligns with the opening of the funnel.
  d. Secure the construction with a cytoclip, and place it into the cytocentrifuge.
  e. Transfer 50 μL of eosinophil suspension into the funnel of the sample chamber.
  f. Centrifuge for 5 min at 500 × *g*, at 19°C–22°C.
  g. Remove the sample chambers from the cytocentrifuge and remove the slides from the setup.
  h. Let the slides dry for 10 min.
    **Pause point:** Store cytospins at −20°C.
  i. Place cytospins into a methanol-filled glass staining trough and fix slides for 5 min.
    **CRITICAL:** Due to the toxicity of methanol, we recommend performing this step under a fume hood.
  j. Allow slides to air dry.
15. Staining cytospins.
  a. Surround the spot where eosinophils are concentrated on the slides with a hydrophobic barrier pen and briefly air dry.
  b. Place cytospins slides on a flat surface.
  c. Pipette 200 μL diluted Giemsa staining solution onto the eosinophil spot.
    ***Note:*** The Giemsa staining solutions should sit on top of the slide in a domed fashion but not overflow over the hydrophobic barrier. It might be necessary to adjust the volume according to the size of the hydrophobic barrier.
  d. Incubate at 19°C–22°C for 20 min.
  e. Wash twice in staining buffer for 1 min each in a staining trough.
  f. Let slides air dry.
  g. Mount slides with 50 μL mounting medium and cover with coverslips.
  h. Let the slides air under the fume hood before starting microscopy analysis.

### Flow cytometry staining to assess CD16 expression on eosinophils

**Timing: 30 min**

This section outlines the procedure for analyzing CD16 expression on eosinophils using flow cytometry. The goal is to emphasize the significance of including CD16+ eosinophils in subsequent experiments. Eosinophils are stained with a CD16 antibody after cell sorting.

16. Centrifuge 50,000 sorted eosinophils for 7 min at 350 × *g* and 4°C in a microcentrifuge.
17. Carefully discard the supernatants without disturbing the cell pellet.
18. Resuspend the eosinophil pellets in 50 μL of PBS and mix thoroughly by pipetting.
19. Add 1.5 μL CD16 antibody and mix thoroughly by pipetting.

***Optional:*** To ensure confident identification of eosinophils when the cells are not completely pure, the eosinophil-specific marker Siglec-8 could also be stained. For that, please keep in mind that the fluorophore-conjugation of the antibodies needs to be adjusted.

20. Incubate cells for 10 min in the dark at 19°C–22°C.
21. Before measurement, dilute the cell suspension with 200 μL of PBS and mix by pipetting.
22. Proceed with flow cytometry analysis.

***Optional:*** If the percentage of CD16 expression is to be analyzed, include an FMO control in order to set the gate for marker positive eosinophils. For that measure eosinophils without staining of CD16 to see where CD16− eosinophils are located.

***Note:*** If exclusively CD16− or CD16+ eosinophils are required for further experiments, eosinophils can potentially be sorted according to their CD16 expression. Set up another sort and stain eosinophils with a CD16 antibody and adjust the sort gate to CD16− or CD16+ eosinophils. However, this procedure has not been tested and verified by us.

### Eosinophil activation test

**Timing: 1 day**

This section describes the procedure to measure the externalization of the eosinophil activation marker CD69 upon stimulation with the eosinophil activating cytokine IL-3.

23. Centrifuge 100,000 eosinophils per condition in a microcentrifuge tube for 7 min and 350 × *g* at 19°C–22°C.
24. Discard the supernatant and resuspend the eosinophil pellet in 90 μL of prewarmed, RPMI 1640 medium with 10% FBS and 1% Penicillin-Streptomycin, for each experimental condition (a total volume of 180 μL with 200,000 cells for two conditions, etc.).
25. Use a 1.5 mL microcentrifuge tube per experimental condition and transfer 90 μL of the eosinophil suspension into each tube.
26. Dilute IL-3 to a concentration of 100 ng/mL.
27. Add 10 μL of the diluted IL-3 to achieve the final concentration of 10 ng/mL in the cell suspension, or RPMI medium for the negative control, and mix well by pipetting.
28. Place the microcentrifuge tubes into a cell incubator and incubate for 21 h at 37°C and 5% CO_2_.

**CRITICAL:** Do not close the lids of the microcentrifuge tubes completely when placing them into the incubator, but leave them slightly open. This allows CO_2_ to reach the eosinophils during incubation. V-bottom 96-well plates may be used as an alternative to microcentrifuge tubes if desired.

29. Place the cells on ice for 2 min to stop cell activity.
30. Centrifuge the cells for 7 min at 350 × *g* and 4°C in a microcentrifuge.
31. Remove and discard the supernatants carefully without disturbing the pellet.
32. Resuspend the eosinophil pellets with 50 μL PBS and mix well by pipetting.
33. Add 1 μL APC-CD69 antibody and mix well by pipetting.

***Optional:*** For eosinophil identification, the eosinophil-specific marker Siglec-8 could also be stained when cells are not completely pure.

34. Incubate cells for 10 min in the dark at 19°C–22°C.
35. Before measurement, dilute the cell suspension with 200 μL of PBS and mix by pipetting.
36. Proceed with flow cytometry analysis. Troubleshooting 5.

***Note:*** To determine cell activation, eosinophils were stained with a CD69 antibody. Marker expression can be either described as percentage of positive eosinophils, or as MFI (mean fluorescence intensity). MFI provides more accurate information about marker expression density on the cell surface, while percentages only give information about if eosinophils express the marker or not.

## Expected outcomes

**Figure 4:**
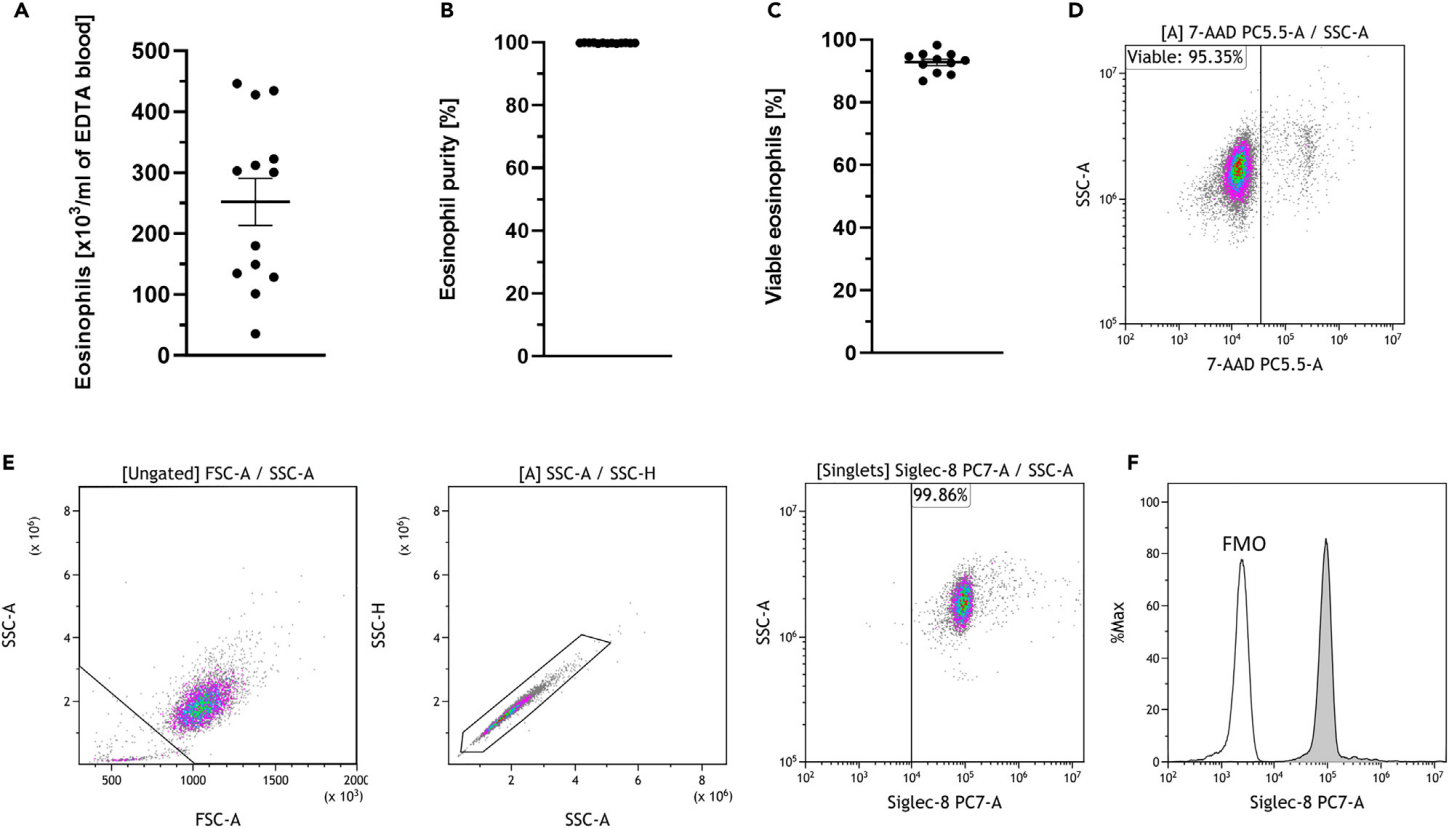
Yield, purity and viability of isolated eosinophils (A) Eosinophil yield was calculated through the number of sorted events and the volume of EDTA blood that was utilized (*n* = 13; ± SEM). (B) Eosinophil purity was assessed by measuring the percentage of Siglec-8 positive cells through flow cytometry (*n* = 13; ± SEM). (C) Eosinophils viability was determined directly after purification through the exclusion of 7-AAD positive eosinophils (*n* = 11; ± SEM). (D) Representative dot plot showing the typical viability of eosinophils directly after purification by measuring the percentage of 7-AAD negative eosinophils. (E) Gating strategy to identify eosinophils through flow cytometry analysis in a representative sample. Side scatter (SSC-A) and forward scatter (FSC-A) properties for detecting eosinophil-sized cells after cell sorting. Eosinophils were identified after the exclusion of doublets through measuring Siglec-8 positive cells. (F) Overlay histogram of the fluorescence minus one (FMO) control and Siglec-8 stained eosinophils.

**Figure 5:**
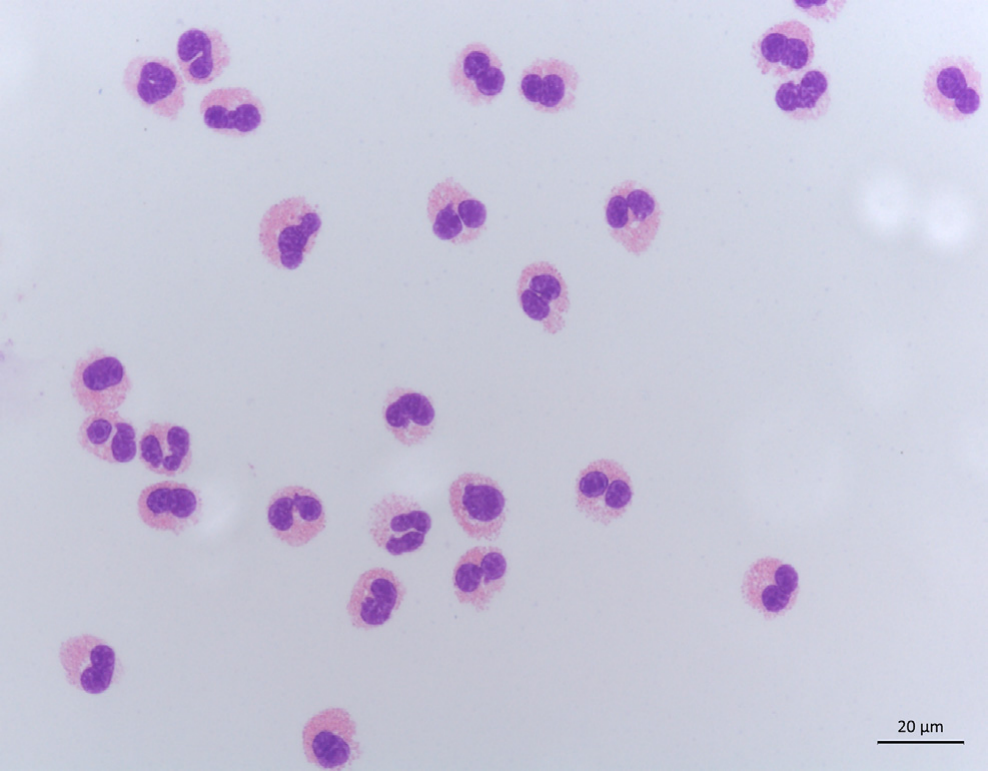
Giemsa staining of isolated human eosinophils Representative bright field microscopy image of eosinophil cytospins at 60x magnification. Eosinophils can be identified by their bilobed nuclei and eosinophilic granules.

We achieved yields between 36,000 and 446,000 eosinophils (mean 252,000/mL) per mL EDTA blood (Figure 4A) with purities consistently exceeding 99% (mean 99.8%; ± 0.03% SEM) (Figure 4B) and a mean viability of 92.8% (± 1.01% SEM) (Figure 4C). The viability was determined by staining with the DNA-intercalating dye 7-AAD. A representative dot plot is shown in Figure 4D. The purity was determined by analyzing singlets and staining with the eosinophil-specific marker Siglec-8. The gate was set according to the fluorescence minus one control (FMO) Figure 4F. Representative dot plots are shown in Figure 4E. Additionally, we visualized eosinophil purity through a microscopic image after Giemsa staining (Figure 5).

**Figure 6:**
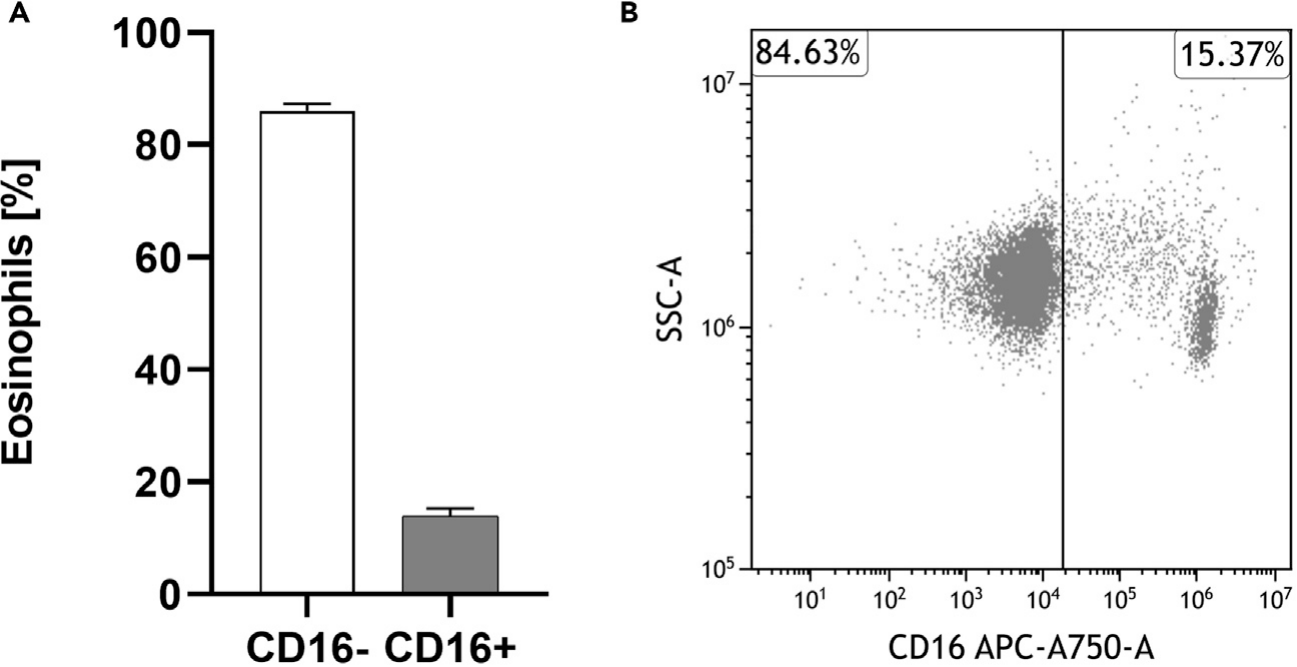
CD16 expression on freshly purified human eosinophils (A) Percentage of CD16− and CD16+ eosinophils directly after isolation determined using flow cytometry (*n* = 13; ± SEM). (B) Representative dot plot demonstrating typical CD16 expression on eosinophils after purification.

Unlike conventional methods, this novel approach also isolates eosinophils that are CD16+. Therefore, we determined its expression through flow cytometry analysis utilizing a CD16 antibody. We found that CD16 is expressed on the cell surface of 14% (± 1.28% SEM) of isolated eosinophils which varies between donors (Figure 6A, *n* = 13). Analyzing CD16+ eosinophils can be crucial when investigating eosinophils in health and disease. For instance, CD16 expression is associated with atopy.^3^ Figure 6B shows a representative dot plot of *n* = 13 different samples of CD16 expression on purified eosinophils.

**Figure 7:**
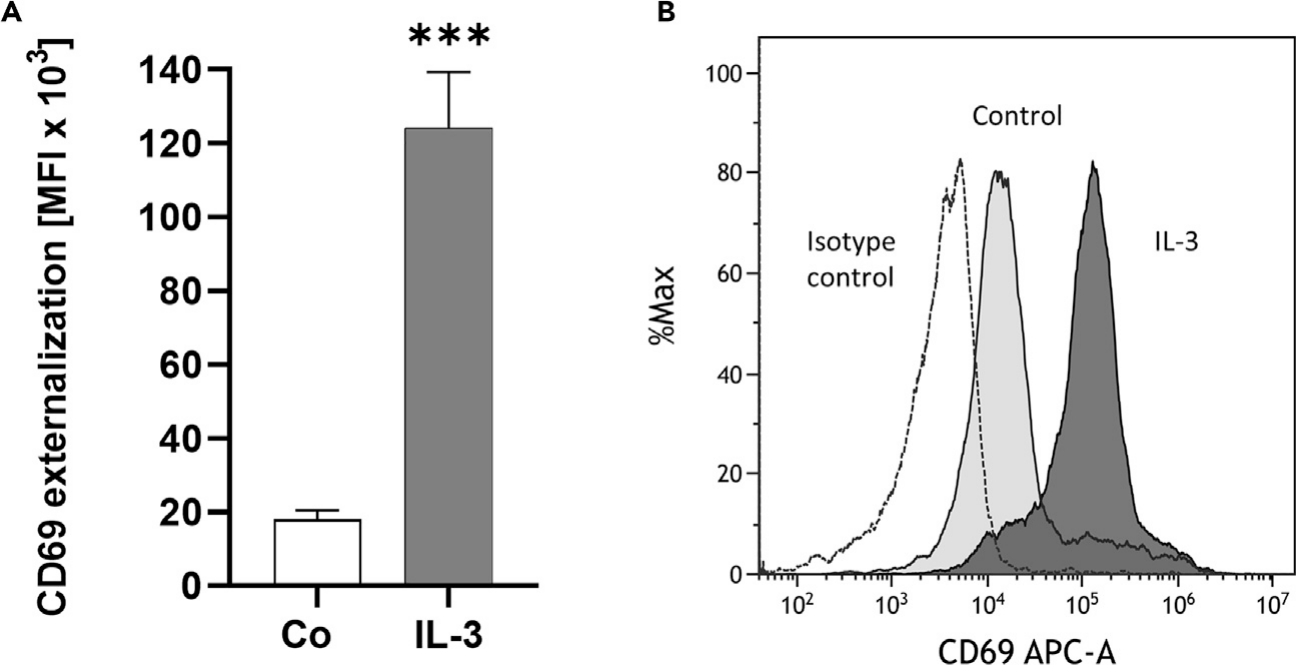
Eosinophil activation test Flow cytometry analysis of CD69 protein externalization after stimulation with IL-3. Eosinophils were stimulated with RPMI medium (Co) or IL-3 (10 ng/mL) for 21 h (*n* = 7). (A) CD69 surface content is significantly increased after stimulation with IL-3. Data shown as mean ± SEM, *p*-values (∗∗∗ ≤ 0.001). Significance is calculated in relation to the Co group. (B) Representative histogram of CD69 MFI of IL-3-stimulated eosinophils, unstimulated eosinophils (control), and CD69 antibody isotype control.

It is critical that eosinophils are not activated during the cell sorting procedure. We therefore conducted a subsequent eosinophil activation test in order to assess their responsiveness to external stimuli. Purified eosinophils were stimulated with IL-3 (10 ng/mL) for 21 h and the surface externalization of the activation marker CD69 was measured. Sorted eosinophils showed low levels of activation which then could be significantly induced by IL-3 (*p* = 0.0003) (Figure 7A). The representative histogram illustrates the shift of CD69 MFI after IL-3 stimulation compared to the negative control and antibody isotype control (Figure 7B). A potential future application of this sorting technique might also be label-free separation of activated eosinophils from non-activated eosinophils through autofluorescence of NADH and NADPH, which is synthetized during cell activation.

## Quantification and statistical analysis

Flow cytometry data were analyzed with the Kaluza software (Beckman Coulter) and GraphPad Prism. A paired *t* test was used for statistical analysis.

## Limitations

When performing this method to purify very high numbers of eosinophils, the yield can be a limiting factor. This limitation arises from natural variations in blood donors, limited sort volume and limited number of cells in suspension that can be sorted precisely. More EDTA blood could be used for purification as long as the abortion rate remains < 1%. Otherwise, a second run on the cell sorter is recommended after all chambers have been emptied and reapplying fresh cell suspension in the input chamber.

## Troubleshooting

### Problem 1

The cartridge has a leak at the bottom and loses liquid during priming, loading, or after starting the sort.

### Potential solution

For priming and loading the cartridge, it is crucial to press the plunger of the syringe slowly and gently. Otherwise the pressure might be too high causing a little crack at the bottom of the cartridge which is often invisible until the sort is started. If a leak is detected after starting the sort, stop the process, remove the cartridge from MACSQuant Tyto Cell Sorter and clean the lens carefully with a dustless paper towel and ethanol. Afterwards, transfer the cell suspension to the input chamber of a new and primed cartridge and restart the sort.

### Problem 2

The cell sorter fails to locate the correct position of the cartridge and stops the process.

### Potential solution

Ensure that the microchip beneath the cartridge remains untouched until sorting begins, and avoid condensation on the cartridge. Any contamination or residue can affect the localization of the correct position. If this occurs, try removing and reinserting the cartridge. If the issue persists, replace the cartridge with a new one.

### Problem 3

The eosinophil purity or yield is poor.

### Potential solution

The cell density may be too high for a precise sorting which could result in poor purity. If the abortion rate exceeds 1% during sorting, the cell sorter is not able to isolate all eosinophils effectively. In this case further dilute the cell suspension in the input chamber and restart the sort. Furthermore, ensure that as much supernatant as possible is decanted after the lysis step to minimize debris.

Additionally, keep in mind that the numbers of eosinophils vary between donors, even within the same donor on different days. In some rare instances, a donor may have complete eosinopenia, making it impossible to isolate eosinophils from their blood samples.

### Problem 4

Eosinophils show poor viability after cell sorting.

### Potential solution

Eosinophils have a relatively short life span of around 18 h in peripheral blood. Thus, blood samples should be cooled and processed within a few hours after collection. We typically always start this protocol within 4 h after blood donation as we have observed that the viability drops to around 80% after 24 h, even when being cooled throughout. After the lysis step the cells should be kept on ice until the sort is started. To ensure low temperatures during the sort, turn on the cell sorter at least half an hour before sorting, as the device needs time to cool down to 4°C. Buffy coats may also be used as a source for eosinophils as we have observed that the viability remains above 90%, even after 24 h in preliminary tests.

### Problem 5

Eosinophils are already activated directly after isolation or in the negative control of the eosinophil activation test.

### Potential solution

The activation status of eosinophils after sorting can strongly differ between different blood donors due to prior contact with substances that the donors might be allergic to. Therefore, CD69 externalization can be increased directly after cell sorting or in the negative control. We recommend performing the eosinophil activation test with blood drawn before exposure to allergens, or with blood from non-atopic donors.

## Resource availability

### Lead contact

Ulrike Raap (raap.ulrike@klinikum-oldenburg.de).

### Technical contact

Tobias Weihrauch (tobias.weihrauch@uni-oldenburg.de).

### Materials availability

This study did not generate new unique reagents.

### Data and code availability

The published article includes all data generated or analyzed during this study.

## Acknowledgments

This work was supported by the German Research Foundation DFG with a grant to U.R. (RA-1026/3-2) (FOR2690-PruSEARCH Translational Pruritus Research). We would like to thank A. Marten and I. Kerkhoff for providing excellent technical support. We also appreciate the assistance of the cell sorting facility supported by the DFG (ID424196510). Furthermore, we thank C. Müller, T. Müller, T. Schricker, K. Schmidt, A. Heineke, N. Windeler, R. Kluck, L. Fieber, I. Latoschinski, and K. Ubber for collecting blood samples from the donors. The authors also thank Arne Knörk from Miltenyi Biotec for sharing his expertise on cell sorting.

## Author contributions

T.W.: conceptualization, data analysis, investigation, methodology, validation, visualization, and writing – original draft. N.G.: investigation, methodology, and writing – review and editing. N.H.M.: conceptualization, methodology, and writing – review and editing. K.L.: writing – review and editing. U.R.: conceptualization, funding acquisition, supervision, validation, and writing – review and editing.

## Declaration of interests

The authors declare no competing interests.
